# Molecular Composition of Plant Vacuoles: Important but Less Understood Regulations and Roles of Tonoplast Lipids

**DOI:** 10.3390/plants4020320

**Published:** 2015-06-11

**Authors:** Chunhua Zhang, Glenn R. Hicks, Natasha V. Raikhel

**Affiliations:** Center for Plant Cell Biology & Department of Botany and Plant Sciences, University of California, 900 University Ave., Riverside, CA 92521, USA; E-Mails: chunhuaz@ucr.edu (C.Z.); glenn.hicks@ucr.edu (G.R.H.)

**Keywords:** vacuole lumen content, tonoplast protein composition, tonoplast lipid composition, vacuolar trafficking

## Abstract

The vacuole is an essential organelle for plant growth and development. It is the location for the storage of nutrients; such as sugars and proteins; and other metabolic products. Understanding the mechanisms of vacuolar trafficking and molecule transport across the vacuolar membrane is of great importance in understanding basic plant development and cell biology and for crop quality improvement. Proteins play important roles in vacuolar trafficking; such proteins include Rab GTPase signaling proteins; cargo recognition receptors; and SNAREs (Soluble NSF Attachment Protein Receptors) that are involved in membrane fusion. Some vacuole membrane proteins also serve as the transporters or channels for transport across the tonoplast. Less understood but critical are the roles of lipids in vacuolar trafficking. In this review, we will first summarize molecular composition of plant vacuoles and we will then discuss our latest understanding on the role of lipids in plant vacuolar trafficking and a surprising connection to ribosome function through the study of ribosomal mutants.

## 1. Introduction

Vacuoles are part of the endomembrane system in plant cells and occupy a large percentage of the cell volume. The vacuolar membrane named the tonoplast separates the cytoplasm from the vacuole lumen. The vacuoles are very dynamic and their morphology changes in response to environmental conditions and varies during different plant developmental stages [1]. Water, ions and metabolic products cannot cross the tonoplast freely without the facilitation of tonoplast proteins. The activities of tonoplast-localized enzymes, transporters and channels change in response to cytoplasmic conditions and regulate material exchange between the cytoplasm and vacuole lumen and maintain cellular homeostasis.

The response of vacuolar transporters to changes in cytoplasmic conditions induced by environmental or cellular signaling is critical for plant growth, nutrient sensing and adaptation to the environment [2]. Characterization and analysis of the regulation of the vacuolar transporters for organic compounds or inorganic ions are important topics in plant biology. In the last decade, proteomics data from vacuoles of different plant species revealed the protein composition of the tonoplast and the vacuole lumen. These data provide candidate targets for studying vacuolar transport and to address the mechanisms of vacuole function. Another important aspect in studying vacuole function is to understand the role of lipids that are integral to the tonoplast. The lipids on the tonoplast provide physical support for proteins and also play roles in regulating tonoplast enzyme activity and vacuole fusion. We have recently provided new evidence on the role of lipids in vacuolar trafficking by studying ribosomal mutants. In this review, we will not cover the important mechanisms of protein vacuolar trafficking pathways; these have been covered recently in other reviews [1,3,4,5,6]. Instead, we will briefly summarize what is known about the content of the vacuole lumen, tonoplast protein and lipid composition in plants. We will then discuss the surprising role of lipids in vacuolar trafficking.

## 2. Plant Vacuole Lumen Content

It has been known for decades that vacuoles are the cellular compartment for storage. This includes proteins, mineral salts, organic acids, amino acids, proteins, sugars, nucleic acids, and glycosides. The static content of the vacuole as well as analysis of dynamic changes in content provide important information for understanding the function of the vacuole in specific cell types. There are several good examples of the known vacuole lumen content in some plant species. Vacuoles isolated from barley mesophyll protoplasts contain both primary and secondary metabolites and are the major site of accumulation of certain amino acids (His, Ala, Trp and Met), sugars and flavonoids [7]. In tobacco cultured cells, excess sulfate and amino acids are transported into the vacuole for storage [8]. In Hippeastrum petal and Tulipa petal and leaves, the majority of the glucose and fructose accumulate in the vacuoles [9]. In oat coleoptiles, where vacuoles occupy around 90% of the cell volume, large amounts of Na^+^, K^+^, and Cl^−^ cross the tonoplast and accumulate in the vacuole [10]. In the storage root of sugar beets (*Beta vulgaris*), most of the Na^+^, K^+^, sucrose and acid invertase for sucrose metabolism accumulate in the vacuoles [11,12]. Detection of the vacuole content in grape subepidermal cells showed that vacuoles in this cell type contain high concentrations of flavonoids, sugar (glucose, fructose and sucrose), organic acids (tartaric, malic, quinic, phosphoric, and citric acids) and cations (K^+^, Ca^2+^, Mg^2+^, Fe^2+,3+^, Na^+^, Al^3+^, Cu^2+^, and Mn^2+^) [13]. Some toxic heavy metals such as cadmium and arsenic also accumulate in vacuoles; this confers the adaptation of some plants to toxic environments [14,15,16]. The distribution of metabolites in vacuoles also fluctuate under different environmental conditions [17]. These data reflect the complexity and diversity of vacuole lumen content and point to the challenges of studying the dynamic control of transport across the tonoplast in response to different cytoplasm conditions.

Besides the contents listed above, some evidence shows that vacuoles are organelles for the accumulation of hormone metabolic products that participate in maintaining cytoplasm hormone homeostasis [18,19,20]. *O*-glucosides of zeatin-type cytokinin accumulate in the vacuole of tobacco, and vacuolar expression of a maize β-glucosidase in tobacco disrupts the level of zeatin-*O*-glucoside, providing evidence for the role of vacuole in maintaining cytokinin level in plants [21]. The gibberellin (GA) metabolite GA_8_-glu is preferentially located in the vacuoles of cowpea and barley leaves fed with [^3^H]GA_1_, indicating that vacuoles are the location for the accumulation of GA_1_ metabolites [22]. One of the major salicylic acid (SA) metabolites, SA 2-*O*-β-d-glucose (SAG) is localized in the vacuoles of tobacco suspension cells [20]. Auxin and its metabolites are also found in plant vacuoles, and auxin transport across the tonoplast plays essential roles in maintaining auxin homeostasis [19]. All these data indicate that vacuoles are not only for the storage of energy reserves; they are involved in the hormone signaling regulation in plants as well.

As in yeast, the plant vacuole is a location for protein degradation. Yeast vacuole lumen proteomics analysis identified different kinds of proteases, indicating that vacuoles function as a lysosomal compartment [23]. Plant lytic vacuoles are also involved in protein turn over and proteomic data from Arabidopsis entire vacuole preparation identified group of proteins involved in protein degradation [24]. Ubiquitinated membrane proteins that are destined for degradation are recognized by receptors and transported to the lytic vacuole for salvage via the late endosome/prevacuolar compartment (PVC) [25,26]. Some known proteins targeted to the vacuole for degradation include important factors in hormone signaling, plant response to pathogen invasion and nutrition sensing [27,28,29,30]. Vacuolar salvage is one way for the cells to control the abundance of these proteins at the cellular location of action. This further confirmed the important roles of vacuolar trafficking in integrating different aspects pathways during plant development and plant response to the environment.

## 3. Proteins Residing at the Tonoplast

The complexity and dynamic changes in vacuole lumen content raise the question of how cells control the flow of these materials between cytosol and the vacuole. Controlled transport across the tonoplast is important for plant responses to environmental conditions or intracellular signaling. Tonoplast membrane proteins can serve to facilitate and regulate biomolecular transport across the membrane. Some vacuolar transporters and channels have been characterized and their regulation of transport across tonoplast has been discussed [2,31,32,33]. In order to identify tonoplast proteins profiles, proteomic analysis of tonoplast preparations from different cell types has been carried out and has revealed similar groups of proteins such as vacuolar ATPases, transporters and proteins involved in membrane fusion [24,34,35,36,37,38,39]. However, nearly 50% of proteins identified from each of these experiments were unidentified in others. This might in part be because the protein composition of the vacuole membrane can vary between different species, cell types or growth conditions. Sensitivity differences among mass spectrometers used in the studies may also contribute to variation in different experiments. Different tonoplast protein composition may cause the diversity of vacuole lumen content. This made it more important to do parallel comparative proteomic experiments to find relative differences among tonoplast proteins involved in specific processes. For example, comparative proteomic analysis identified more than 10 vacuolar membrane proteins that are changed upon gibberellin treatment and revealed that the glycolytic pathway is coupled to the V-ATPase proton pump to mediate root growth [40]. Also, quantitative proteomic analysis from barley plants treated with different concentrations of cadmium resulted in the identification of proteins that are elevated in response to cadmium treatment. Such proteins provided good candidates for studying cadmium detoxification mediated by the vacuole [41]. These data confirmed that tonoplast proteins could be regulated by physiological and environmental conditions. Additional comparative analyses could provide more information aimed at understanding the functions of key tonoplast proteins in important cellular process.

## 4. Tonoplast Lipid Composition, Distribution and Function

Lipids in the tonoplast provide a molecular environment for membrane proteins and serve as the barrier between the cytoplasm and the vacuole lumen. Membrane curvature and fluidity, which are determined by the membrane lipid composition, are important factors in vacuolar fusion [42]. In yeast, different groups of lipids such as phosphoinositides, ergosterol, diacylglyceol (DAG), phosphatidic acid (PA) and phosphatidylethanolamine (PE) are required for vacuole fusion and are critical for proper vacuole biogenesis [43]. In plant, phosphoinositides are critical for vacuole biogenesis, although the mechanisms for their action need further investigation [1,44,45,46]. Lipids also affect the enzymatic activities of vacuolar proteins. For example, sphingolipids in yeast participate in regulating the activities of V-ATPase necessary for normal vacuole acidification [47]. Thus, vacuole membrane lipids are essential for maintaining proper vacuole function but the mechanisms of their function and regulation are less understood in plants.

The lipid composition of the tonoplast varies between different species. In the tonoplast of etiolated mung bean (*Vigna radiata* L.) hypocotyls, 51% of the total lipids are phospholipids, 27.9% are sterols and ceramide monohexoside composes 16.6% of the total lipids [48]. Mono- and digalactosyldiglycerides were also found in the mung bean tonoplast. In sycamore (*Acer pseudoplatanus*) cultured cells, the phospholipids account for 44.5% of the total lipids and sterols account for 30.8% of the total lipids [49]. In yeast (*Saccharomyces cerevisiae*) vacuole membrane, the majority (about 90%) of the total lipids was found to be phospholipids, and a small fraction (about 10%) of ergosterols [50]. Plant cell vacuole membrane has higher percentage of sterols than yeast. The variations in the vacuole membrane lipid compositions may reflect specialized functions and regulation of the vacuole in different species and kingdoms.

Phosphatidylcholine (PC) and PE are dominant phospholipids in the vacuole membrane of both plant and yeast; they are followed by phosphatidylinositol (PI) (Table 1). The contents of phosphatidylserine (PS), phosphatidylglycerol (PG) and PA are low, except in pineapple fruit that has high PA level. This could be a consequence of lipid catabolism during post-harvest storage. In yeast, vacuoles contain higher percentage of PC and lower percentage of PS than the plasma membrane [50]. This significant composition difference was not found in etiolated mung bean hypocotyles [48]. The fatty acid composition of tonoplast lipids also varies between different species, but a high content of unsaturated fatty acids were found in the tonoplast of carrot, red beet and garden radish roots [51]. The tonoplast lipid composition changes under different temperature. High temperature treatment significantly reduces tonoplast phospholipid content in the leaves of pineapple (*Ananas comosus*) and *Kalanchoe* *pinnata* [52,53]. Under low temperature (10 °C for a week), an absolute vacuolar lipid content in pineapple fruit is significantly reduced. The percentage of PE and PC in total lipids are also reduced and the percentage of PA in total lipids is increased [54]. Changes in the tonoplast lipid composition under different temperatures may contribute to this species exquisite adaptation to the environment.

**Table 1:** Phospholipids composition (% mole of total phospholipids) of vacuoles from different species.

	Pineapple Fruit ^a^	Mung Bean Hypocotyls ^b^	Sycamore Cultured Cells ^c^	Yeast ^d^
PC	50.4	46.4	31.9	46.5
PE	24.6	31.3	46.7	19.4
PG	2.1	4.5	2.3	NA
PI	6.6	11.1	15.3	18.3
PS	2.7	4.3	2.5	4.4
PA	13.7	2.2	1.4	2.1
CL	NA	NA	NA	1.6

PC: phosphatidylcholine; PE: phosphatidylethanolamine; PG: phosphatidylglycerol; PI: phosphatidylinositol; PS: phosphatidylserine; PA: phosphatidic acid; CL: cardiolipin; NA: not available; ^a^: Data from Zhou *et al.* [54]; ^b^: Data from Yoshida and Uemura [48]; ^c^: Data from Tavernier *et al.* [49]; ^d^: Data from Zinster *et al.* [50].

PVC is a source of some vacuole membrane lipids. PC in the yeast vacuole membrane is imported from the PVC, and a vacuolar localized ABC transporter Ybt1 is required for this import [55,56]. Phosphatidylinositide-3-phosphate (PI3P) is localized to the vacuole membrane and late endosome/PVC in plant cells [57,58]. PI3P enters the vacuole through the PVC as well because a transiently expressed fluorescent marker for PI3P was found in the trans-Golgi network, the PVC, the vacuole membrane, and vesicles within the vacuole lumen in a time-course manner. Although direct protein trafficking from endoplasmic reticulum (ER) to the vacuole, bypassing the Golgi and post-Golgi compartments, has been shown in plants [59,60,61,62,63], there is no evidence shows direct lipids import from ER to the vacuole in plants.

Lipids and proteins are not evenly distributed on the vacuole membrane. In yeast cells at the stationary phase, there are microdomains on the vacuoles that are enriched in sterols and vacuolar proteins are segregated by these microdomains [64]. The formation of these special microdomains is regulated by glucose, pH-responsive pathway and vacuolar trafficking pathway. The sterol-enriched vacuolar microdomains in yeast mediate the transport of lipid drops from the perinuclear endoplasmic reticulum to the vacuole during the transition to stationary phase [65]. In Arabidopsis cultured cells, the vacuoles also contain microdomains with higher ratios of saturated fatty acids in phospholipids PC and PE [66]. Mass Spectrometry analysis showed that vacuolar proteins have different localization patterns in these microdomains. For example, the vacuolar-type proton ATPase (V-ATPase) was more enriched in detergent-resistant microdomains but the vacuolar-type proton pyrophosphatase (V-PPase) was distributed more evenly in different membrane fractions. These data provided new insights in the mechanisms of vacuolar transport and vacuolar trafficking. In sugarbeet roots, there are also detergent-resistant microdomains on the tonoplast that contain a high percentage of sphingolipids, free sterols and saturated fatty acids [67]. The exact roles of these microdomains in sugarbeet are not clear, but it would be interesting to identify proteins that accumulate in these microdomains and determine whether they are related to sucrose accumulation in root cell vacuoles [66]. The precise distribution of lipids and proteins at the tonoplast can be regulated by environmental conditions. For example, under phosphate deficiency, Arabidopsis phospholipase D PLDζ2 is concentrated at specific loci on the tonoplast and my represent tonoplast domains for active phospholipid catabolism [68]. There is also an asymmetric distribution of lipids on the two membrane leaflets of the tonoplast. About 20% more PE is distributed in outside lipid monolayer compares with the inner monolayer, whereas PC has the same distribution in both leaflets of the lipid bilayer in the tonoplast of *Acer pseudoplatanus* cells [69]. Complex composition, dynamic change and uneven and asymmetric distribution of lipids and proteins at tonoplasts reflect dynamic regulation of vacuolar transport.

Phospholipid catabolism can occur on tonoplast. Both phospholipase A and phospholipase D activities have been identified in tonoplast preparations [70]. Loss of function of tonoplast localized Arabidopsis PLDζ2 results in membrane accumulation within the vacuole [68]. As mentioned above, phosphate deficiency induced PLDζ2 redistribution at the tonoplast, and it indicates that vacuole lipid metabolism can be regulated by environmental conditions.

The phospholipids on the route to the vacuole are not simply targeted for degradation. Rather, they play roles in regulating vesicle trafficking processes. Different types of phosphorylated phosphatidylinositol lipids (PPIs) have been shown to involve in plant vacuole morphology and vacuolar trafficking [1]. Reduced availability of PI3P by overexpression of a lipid binding protein inhibited protein trafficking to the vacuole [58]. A very recent genetic study further confirmed the role of lipids in the plant vacuolar trafficking pathway [71]. A new plant ESCRT (endosomal sorting complex required for transport) component FREE1 was identified as a PI3P interacting protein using its FYVE domain. FREE1 interacts with ubiquitin and is required for the sorting of ubiquitinated membrane cargo protein to the vacuole for degradation. Phosphatidylinositol 3,5-biphosphate (PI(3,5)P_2_) is required for abscisic acid (ABA) induced vacuole acidification and vacuole convoluton in guard cells [44]. The phosphatases that catalyze the decomposition of PI(3,5)P_2_ are localized to the tonoplast and manipulating the level of these enzymes affects both vacuolar trafficking and vacuole morphology, further confirming the important roles and complicate regulation of PPIs in vacuole function [45]. PI(3,5)P_2_ interacts with vacuolar proton pyrophosphatase (V-PPase) directly *in vitro* [44]. It is possible that this lipid-protein interaction is one way for plants to regulate vacuole acidification in response to environmental conditions and growth cues. These examples show that PI3P is an essential lipid in vacuolar trafficking pathway and the metabolism of PPIs along the vacuolar trafficking route provides different forms of PPIs that interact with vacuolar proteins to regulate their enzymatic activities.

## 5. Ribosomal Mutants Reveal the Role of Lipids in Vacuolar Trafficking

Plant growth and development involve multiple cellular processes including hormone signaling, vesicle trafficking, cell wall assembly, protein translational and post-translational regulation, and lipid biosynthesis and degradation. Our recent efforts in dissecting the mechanisms of ribosomal proteins in regulating vacuolar trafficking showed that vacuolar trafficking is one of the hubs that integrate these multiple processes.

The regulatory role of ribosomal proteins in vacuolar trafficking was first identified as a result of a screen for novel vacuolar trafficking regulators using a T-DNA-mutagenized population in Arabidopsis [72]. The mutant *rpl4a*, which has T-DNA insertion in a large 60S ribosomal complex subunit RPL4A (ribosomal protein L4A), causes the redirection of trafficking of marker proteins with a vacuolar sorting signal to the default secretion pathway rather than the vacuole. Further investigation on the mechanisms of RPL4 regulation of vacuolar trafficking showed that proteins in multiple pathways are coordinately regulated at the translational level, and these pathways are involved in vacuolar trafficking.

The auxin-related developmental phenotypes in *rpl4* mutants indicated a possible linkage between ribosome biogenesis, auxin signaling and vacuolar trafficking. It was found that ribosome proteins regulate the translation of multiple auxin response factors (ARFs) and PIN auxin transporters [73]. The abundance of ARFs and PINs is reduced in *rpl* mutants, and this resulted in less auxin sensitivity in these mutants. Auxin signaling affects vacuolar trafficking of different cargos, and the reduced levels of ARFs and PINs and the reduced auxin sensitivity may contribute to the abnormal vacuolar trafficking phenotypes in *rpl* mutants [72,73].

In order to identify other possible vacuolar trafficking regulators that are under the control of ribosome proteins, a systems approach was taken to identify mRNAs that have reduced translational efficiency in *rpl4d* [74]. Quantification of the abundance of ribosome-bound mRNA in wildtype and *rpl4d* showed a group of genes whose translation was down regulated in *rpl4d*. Unsurprisingly, the abundance of polysome-bound mRNA from a total of 1800 genes was reduced in *rpl4d*, reflecting the translational regulation of these genes. However, it was quite surprising that the largest group of genes that has reduced translational efficiency was involved in lipid metabolic processes. Although lipid content quantification using Mass Spectrometry analysis did not reveal significant difference in the amount of lipids of different headgroups in entire young seedlings of *rpl4d*, fluorescence dye labeling showed reduced levels of lipids in the *rpl4d* mutant roots. It is possible that the lipids are abnormally distributed within the cells in *rpl4d* and the difference could not be detected at the whole seedling level. Lipid biosynthesis inhibitor treatment of Arabidopsis roots results in the secretion of a fluorescent protein containing a vacuole-sorting signal. Mutants in lipid biosynthesis genes show similar vacuolar trafficking defects as the lipid inhibitor treatments. These data suggest that lipid metabolism pathways are involved in vacuolar trafficking downstream of translational regulation, and the lipid metabolic deficiency in *rpl4d* at least partially causes vacuolar trafficking defects.

Besides lipid metabolic genes, there are other genes that have reduced translation efficiency in *rpl4d* seedlings. These genes include those involved in the metabolism of amino acids, aromatic compounds, phenylpropanoids, flavonoids, and in endomembrane trafficking. There is a possibility that reduced vacuolar trafficking resulted from down regulation of multiple metabolic pathways. It is also possible that down regulation of special vacuolar trafficking machinery proteins or vacuolar lipids result in the vacuolar trafficking defects. Further quantitative proteomic and lipidomic data analysis from vacuoles of ribosome mutants will reveal direct regulatory components in vacuolar trafficking. In either case, the discovery that ribosomal protein can regulate vacuolar trafficking showed that vacuolar trafficking is part of a complex regulatory network for plant development that involves lipid metabolism, hormone signaling, and protein translational regulation.

## 6. State of the Field, Challenges, and Future Prospects

It is obvious that lipids are important for proper vacuole function in plants. However, our understanding of the exact roles of each lipid is very limited. It is not clear which lipids are required for vacuole fusion and which lipids regulate the activities of vacuole transporters or channels. The basic facts on vacuole membrane lipid composition and distribution in different species set the foundations for further investigation. However, lipids are essential for function of the entire endomembrane system and manipulation of lipid composition by genetic modification of essential genes would be predicted to affect general membrane biology rather than vacuole function only. An *in vitro* vacuole fusion system like that was developed in yeast will address the roles of each lipid in plant vacuole fusion without disrupting the entire endomembrane system. Another challenge is that it is difficult to label certain lipids to study their dynamics and distribution in the tonoplast. Fluorescence-tagged specific lipid-binding domains are useful probes to label special lipids *in vivo*. However, constitutive expression of a strong lipid-binding motif can compete with endogenous lipid-binding proteins and thus can have a negative effect on lipid function. Transient expression of the probes can partially overcome this problem, as shown for PI(3)P dynamics using a GFP fusion probe [58]. Small molecular weight fluorescence lipid-binding dyes could also be an approach to label tonoplast lipids and may make it possible to study their roles in vacuole function.

Some lipids and proteins are concentrated on detergent resistant microdomains of the tonoplast and the dynamics of these domains are under the regulation of cytoplasmic conditions. It is important to understand how different lipids create membrane environments and how they regulate the activities of different tonoplast proteins in response to different signals. A first step could be to detect the protein and lipid composition of these microdomains using mass spectrometry. It won’t be surprising if different plant species have variations in the composition of tonoplast microdomains, as this might relate to specialized functions of vacuoles. With the progress in characterizing the functions of vacuole membrane proteins, *in vitro* biochemical assays can be used to test whether their activities are under regulation by vacuole lipids.

Vacuolar trafficking defects and altered lipid metabolism profile in ribosomal mutants indicated the roles of lipids in vacuolar trafficking. It is surprising that more lipid metabolic genes are affected than vesicle trafficking genes in *rpl4d* and that a lipid biosynthesis gene can partially rescue vacuole trafficking defects. It is not known whether the lipid metabolism defects affect the tonoplast lipid composition or the upstream vacuolar trafficking pathway. To get further mechanistic knowledge of lipid function in vacuolar trafficking, we can compare the tonoplast proteomic and lipidomic profiles of ribosome mutants to that of wildtype. This can overcome the complexity of RNAseq data and provide direct candidates related to vacuole function.

After vacuole fusion, the lipids in the PVC either become part of the tonoplast or are internalized to the vacuole lumen for decomposition. It is not clear how the cells balance the distribution of lipids in two locations. During fast cell growth, lipids are required for tonoplast expansion to increase the cell volume. While during stress conditions, more lipids might be decomposed to provide required energy or nutrition to cope with the stress. Tonoplast lipid redistribution might be required for dynamic vacuole morphology changes such as vacuole convolution during stomatal closure. The mechanisms for lipid remodeling during these processes require further investigation.

Because of the storage function of the vacuoles, an understanding of tonoplast lipid and protein dynamics, mechanisms of vacuolar trafficking, and transport of material across the tonoplast have potential agricultural applications. Crop yield and nutritional value improvement can be realized by regulating vacuolar trafficking and transport steps. Crop adaptation to adverse environmental conditions such as drought, high salt and toxic metals relates to special regulation at the vacuole. A systems approach to compare tonoplast lipid and protein dynamics between different crop varieties or during stress challenge may reveal vacuolar components that can be manipulated to improve agricultural traits.

## References

[1] Zhang C., Hicks G.R., Raikhel N.V. (2014). Plant vacuole morphology and vacuolar trafficking. Front. Plant Sci..

[2] Martinoia E., Meyer S., de Angeli A., Nagy R. (2012). Vacuolar transporters in their physiological context. Annu. Rev. Plant Biol..

[3] Pedrazzini E., Komarova N.Y., Rentsch D., Vitale A. (2013). Traffic routes and signals for the tonoplast. Traffic.

[4] Rojas-Pierce M. (2013). Targeting of tonoplast proteins to the vacuole. Plant Sci..

[5] Uemura T., Ueda T. (2014). Plant vacuolar trafficking driven by rab and snare proteins. Curr. Opin. Plant Biol..

[6] Xiang L., Etxeberria E., van den Ende W. (2012). Vacuolar protein sorting mechanisms in plants. FEBS J..

[7] Tohge T., Ramos M.S., Nunes-Nesi A., Giavalisco P., Steinhauser D., Schellenberg M., Mutwil M., Willmitzer L., Persson S., Martinoia E. (2011). Toward the storage metabolome: Profiling the barley vacuole. Plant Physiol..

[8] Smith I.K. (1981). Compartmentation of sulfur metabolites in tobacco cells: Use of efflux analysis. Plant Physiol..

[9] Wagner G.J. (1979). Content and vacuole/extravacuole distribution of neutral sugars, free amino acids, and anthocyanin in protoplasts. Plant Physiol..

[10] Pierce W.S., Higinbotham N. (1970). Compartments and fluxes of K, Na, and Cl in avena coleoptile cells. Plant Physiol..

[11] Leigh R.A., Rees T., Fuller W.A., Banfield J. (1979). The location of acid invertase activity and sucrose in the vacuoles of storage roots of beetroot (*Beta vulgaris*). Biochem. J..

[12] Leigh R.A., Tomos A.D. (1983). An attempt to use isolated vacuoles to determine the distribution of sodium and potassium in cells of storage roots of red beet (*Beta vulgaris* L*.*). Planta.

[13] Moskowitz A.H., Hrazdina G. (1981). Vacuolar contents of fruit subepidermal cells from vitis species. Plant Physiol..

[14] Guo J., Xu W., Ma M. (2011). The assembly of metals chelation by thiols and vacuolar compartmentalization conferred increased tolerance to and accumulation of cadmium and arsenic in transgenic *Arabidopsis thaliana*. J. Hazard. Mater..

[15] Fu X., Dou C., Chen Y., Chen X., Shi J., Yu M., Xu J. (2010). Subcellular distribution and chemical forms of cadmium in phytolacca americana l. J. Hazard. Mater..

[16] Yang X., Chen H., Dai X., Xu W., He Z., Ma M. (2009). Evidence of vacuolar compartmentalization of arsenic in the hyperaccumulator *Pteris vittata*. Chin. Sci. Bull..

[17] Oikawa A., Matsuda F., Kikuyama M., Mimura T., Saito K. (2011). Metabolomics of a single vacuole reveals metabolic dynamism in an alga chara australis. Plant Physiol..

[18] Kulich I., Zarsky V. (2014). Autophagy-related direct membrane import from er/cytoplasm into the vacuole or apoplast: A hidden gateway also for secondary metabolites and phytohormones?. Int. J. Mol. Sci..

[19] Ranocha P., Dima O., Nagy R., Felten J., Corratge-Faillie C., Novak O., Morreel K., Lacombe B., Martinez Y., Pfrunder S. (2013). Arabidopsis WAT1 is a vacuolar auxin transport facilitator required for auxin homoeostasis. Nat. Commun..

[20] Dean J.V., Mohammed L.A., Fitzpatrick T. (2005). The formation, vacuolar localization, and tonoplast transport of salicylic acid glucose conjugates in tobacco cell suspension cultures. Planta.

[21] Kiran N.S., Benkova E., Rekova A., Dubova J., Malbeck J., Palme K., Brzobohaty B. (2012). Retargeting a maize beta-glucosidase to the vacuole—Evidence from intact plants that zeatin-*O*-glucoside is stored in the vacuole. Phytochemistry.

[22] Garcia-Martinez J.L. (1981). Differential compartmentation of gibberellin A(1) and its metabolites in vacuoles of cowpea and barley leaves. Plant Physiol..

[23] Sarry J.E., Chen S., Collum R.P., Liang S., Peng M., Lang A., Naumann B., Dzierszinski F., Yuan C.X., Hippler M. (2007). Analysis of the vacuolar luminal proteome of *Saccharomyces cerevisiae*. FEBS J..

[24] Carter C., Pan S., Zouhar J., Avila E.L., Girke T., Raikhel N.V. (2004). The vegetative vacuole proteome of *Arabidopsis thaliana* reveals predicted and unexpected proteins. Plant Cell.

[25] Scheuring D., Kunzl F., Viotti C., Yan M.S., Jiang L., Schellmann S., Robinson D.G., Pimpl P. (2012). Ubiquitin initiates sorting of golgi and plasma membrane proteins into the vacuolar degradation pathway. BMC Plant Biol..

[26] Herberth S., Shahriari M., Bruderek M., Hessner F., Muller B., Hulskamp M., Schellmann S. (2012). Artificial ubiquitylation is sufficient for sorting of a plasma membrane atpase to the vacuolar lumen of arabidopsis cells. Planta.

[27] Bayle V., Arrighi J.F., Nespoulous C., Vialaret J., Rossignol M., Gonzalez E., Paz-Ares J., Creff A., Nussaume L. (2011). *Arabidopsis thaliana* high-affinity phosphate transporters exhibit multiple levels of posttranslational regulation. Plant Cell.

[28] Abas L., Benjamins R., Malenica N., Paciorek T., Wisniewska J., Moulinier-Anzola J.C., Sieberer T., Friml J., Luschnig C. (2006). Intracellular trafficking and proteolysis of the Arabidopsis auxin-efflux facilitator pin2 are involved in root gravitropism. Nat. Cell Biol..

[29] Kleine-Vehn J., Leitner J., Zwiewka M., Sauer M., Abas L., Luschnig C., Friml J. (2008). Differential degradation of pin2 auxin efflux carrier by retromer-dependent vacuolar targeting. Proc. Natl. Acad. Sci. USA.

[30] Altenbach D., Robatzek S. (2007). Pattern recognition receptors: From the cell surface to intracellular dynamics. Mol. Plant Microbe Interact..

[31] Neuhaus H.E., Trentmann O. (2014). Regulation of transport processes across the tonoplast. Front. Plant Sci..

[32] Hedrich R. (2012). Ion channels in plants. Physiol. Rev..

[33] Isayenkov S., Isner J.C., Maathuis F.J. (2010). Vacuolar ion channels: Roles in plant nutrition and signalling. FEBS Lett..

[34] Endler A., Meyer S., Schelbert S., Schneider T., Weschke W., Peters S.W., Keller F., Baginsky S., Martinoia E., Schmidt U.G. (2006). Identification of a vacuolar sucrose transporter in barley and *Arabidopsis* mesophyll cells by a tonoplast proteomic approach. Plant Physiol..

[35] Jaquinod M., Villiers F., Kieffer-Jaquinod S., Hugouvieux V., Bruley C., Garin J., Bourguignon J. (2007). A proteomics dissection of *Arabidopsis thaliana* vacuoles isolated from cell culture. Mol. Cell. Proteomics.

[36] Trentmann O., Haferkamp I. (2013). Current progress in tonoplast proteomics reveals insights into the function of the large central vacuole. Front. Plant Sci..

[37] Shimaoka T., Ohnishi M., Sazuka T., Mitsuhashi N., Hara-Nishimura I., Shimazaki K., Maeshima M., Yokota A., Tomizawa K., Mimura T. (2004). Isolation of intact vacuoles and proteomic analysis of tonoplast from suspension-cultured cells of *Arabidopsis thaliana*. Plant Cell. Physiol..

[38] Schmidt U.G., Endler A., Schelbert S., Brunner A., Schnell M., Neuhaus H.E., Marty-Mazars D., Marty F., Baginsky S., Martinoia E. (2007). Novel tonoplast transporters identified using a proteomic approach with vacuoles isolated from cauliflower buds. Plant Physiol..

[39] Szponarski W., Sommerer N., Boyer J.C., Rossignol M., Gibrat R. (2004). Large-scale characterization of integral proteins from arabidopsis vacuolar membrane by two-dimensional liquid chromatography. Proteomics.

[40] Konishi H., Maeshima M., Komatsu S. (2005). Characterization of vacuolar membrane proteins changed in rice root treated with gibberellin. J. Proteome Res..

[41] Schneider T., Schellenberg M., Meyer S., Keller F., Gehrig P., Riedel K., Lee Y., Eberl L., Martinoia E. (2009). Quantitative detection of changes in the leaf-mesophyll tonoplast proteome in dependency of a cadmium exposure of barley (*Hordeum vulgare* L.) plants. Proteomics.

[42] Karunakaran S., Fratti R.A. (2013). The lipid composition and physical properties of the yeast vacuole affect the hemifusion-fusion transition. Traffic.

[43] Wickner W. (2010). Membrane fusion: Five lipids, four snares, three chaperones, two nucleotides, and a rab, all dancing in a ring on yeast vacuoles. Annu. Rev. Cell Dev. Biol..

[44] Bak G., Lee E.J., Lee Y., Kato M., Segami S., Sze H., Maeshima M., Hwang J.U. (2013). Rapid structural changes and acidification of guard cell vacuoles during stomatal closure require phosphatidylinositol 3,5-bisphosphate. Plant Cell.

[45] Novakova P., Hirsch S., Feraru E., Tejos R., van Wijk R., Viaene T., Heilmann M., Lerche J., de Rycke R., Feraru M.I. (2014). Sac phosphoinositide phosphatases at the tonoplast mediate vacuolar function in Arabidopsis. Proc. Natl. Acad. Sci. USA.

[46] Whitley P., Hinz S., Doughty J. (2009). Arabidopsis fab1/pikfyve proteins are essential for development of viable pollen. Plant Physiol..

[47] Finnigan G.C., Ryan M., Stevens T.H. (2011). A genome-wide enhancer screen implicates sphingolipid composition in vacuolar atpase function in saccharomyces cerevisiae. Genetics.

[48] Yoshida S., Uemura M. (1986). Lipid composition of plasma membranes and tonoplasts isolated from etiolated seedlings of mung bean (*Vigna radiata* L.). Plant Physiol..

[49] Tavernier E., le Quoc D., le Quoc K. (1993). Lipid composition of the vacuolar membrane of acer pseudoplatanus cultured cells. Biochim. Biophys. Acta.

[50] Zinser E., Sperka-Gottlieb C.D., Fasch E.V., Kohlwein S.D., Paltauf F., Daum G. (1991). Phospholipid synthesis and lipid composition of subcellular membranes in the unicellular eukaryote saccharomyces cerevisiae. J. Bacteriol..

[51] Makarenko S.P., Konenkina T.A., Salyaev R.K. (2000). Characteristics of fatty acid composition of lipids in higher plant vacuolar membranes. Membr. Cell Biol..

[52] Lin Q., Wang Y.M., Nose A., Hong H.T.K., Agarie S. (2008). Effects of high night temperature on lipid and protein compositions in tonoplasts isolated from ananas comosus and *Kalanchoë pinnata* leaves. Biol. Plant..

[53] Behzadipour M., Ratajczak R., Faist K., Pawlitschek P., Tremolieres A., Kluge M. (1998). Phenotypic adaptation of tonoplast fluidity to growth temperature in the cam plant *Kalanchoe daigremontiana* ham. Et per. Is accompanied by changes in the membrane phospholipid and protein composition. J. Membr. Biol..

[54] Zhou Y., Pan X., Qu H., Underhill S.J. (2014). Tonoplast lipid composition and proton pump of pineapple fruit during low-temperature storage and blackheart development. J. Membr. Biol..

[55] Hanson P.K., Grant A.M., Nichols J.W. (2002). Nbd-labeled phosphatidylcholine enters the yeast vacuole via the pre-vacuolar compartment. J. Cell Sci..

[56] Gulshan K., Moye-Rowley W.S. (2011). Vacuolar import of phosphatidylcholine requires the atp-binding cassette transporter ybt1. Traffic.

[57] Simon M.L., Platre M.P., Assil S., van Wijk R., Chen W.Y., Chory J., Dreux M., Munnik T., Jaillais Y. (2013). A multi-colour/multi-affinity marker set to visualize phosphoinositide dynamics in Arabidopsis. Plant J..

[58] Kim D.H., Eu Y.J., Yoo C.M., Kim Y.W., Pih K.T., Jin J.B., Kim S.J., Stenmark H., Hwang I. (2001). Trafficking of phosphatidylinositol 3-phosphate from the trans-golgi network to the lumen of the central vacuole in plant cells. Plant Cell.

[59] Batistic O., Waadt R., Steinhorst L., Held K., Kudla J. (2009). CBL-mediated targeting of CIPKs facilitates the decoding of calcium signals emanating from distinct cellular stores. Plant J..

[60] Bottanelli F., Foresti O., Hanton S., Denecke J. (2011). Vacuolar transport in tobacco leaf epidermis cells involves a single route for soluble cargo and multiple routes for membrane cargo. Plant Cell.

[61] De Marchis F., Bellucci M., Pompa A. (2013). Traffic of human alpha-mannosidase in plant cells suggests the presence of a new endoplasmic reticulum-to-vacuole pathway without involving the golgi complex. Plant Physiol..

[62] Gomez L., Chrispeels M.J. (1993). Tonoplast and soluble vacuolar proteins are targeted by different mechanisms. Plant Cell.

[63] Viotti C., Kruger F., Krebs M., Neubert C., Fink F., Lupanga U., Scheuring D., Boutte Y., Frescatada-Rosa M., Wolfenstetter S. (2013). The endoplasmic reticulum is the main membrane source for biogenesis of the lytic vacuole in Arabidopsis. Plant Cell.

[64] Toulmay A., Prinz W.A. (2013). Direct imaging reveals stable, micrometer-scale lipid domains that segregate proteins in live cells. J. Cell Biol..

[65] Wang C.W., Miao Y.H., Chang Y.S. (2014). A sterol-enriched vacuolar microdomain mediates stationary phase lipophagy in budding yeast. J. Cell Biol..

[66] Yoshida K., Ohnishi M., Fukao Y., Okazaki Y., Fujiwara M., Song C., Nakanishi Y., Saito K., Shimmen T., Suzaki T. (2013). Studies on vacuolar membrane microdomains isolated from arabidopsis suspension-cultured cells: Local distribution of vacuolar membrane proteins. Plant Cell Physiol..

[67] Ozolina N.V., Nesterkina I.S., Kolesnikova E.V., Salyaev R.K., Nurminsky V.N., Rakevich A.L., Martynovich E.F., Chernyshov M.Y. (2013). Tonoplast of beta vulgaris l. Contains detergent-resistant membrane microdomains. Planta.

[68] Yamaryo Y., Dubots E., Albrieux C., Baldan B., Block M.A. (2008). Phosphate availability affects the tonoplast localization of pldzeta2, an *Arabidopsis thaliana* phospholipase d. FEBS Lett..

[69] Tavernier E., Pugin A. (1995). Transbilayer distribution of phosphatidylcholine and phosphatidylethanolamine in the vacuolar membrane of acer pseudoplatanus cells. Biochimie.

[70] Tavernier E., Pugin A. (1995). Phospholipase activities associated with the tonoplast from acer pseudoplatanus cells: Identification of a phospholipase a1 activity. Biochim. Biophys. Acta.

[71] Gao C., Luo M., Zhao Q., Yang R., Cui Y., Zeng Y., Xia J., Jiang L. (2014). A unique plant escrt component, free1, regulates multivesicular body protein sorting and plant growth. Curr. Biol..

[72] Rosado A., Sohn E.J., Drakakaki G., Pan S., Swidergal A., Xiong Y., Kang B.H., Bressan R.A., Raikhel N.V. (2010). Auxin-mediated ribosomal biogenesis regulates vacuolar trafficking in Arabidopsis. Plant Cell.

[73] Rosado A., Li R., van de Ven W., Hsu E., Raikhel N.V. (2012). Arabidopsis ribosomal proteins control developmental programs through translational regulation of auxin response factors. Proc. Natl. Acad. Sci. USA.

[74] Li R., Sun R., Hicks G.R., Raikhel N.V. (2014). *Arabidopsis* ribosomal proteins control vacuole trafficking and developmental programs through the regulation of lipid metabolism. Proc. Natl. Acad. Sci. USA.

